# Enhanced Sinterability, Thermal Conductivity and Dielectric Constant of Glass-Ceramics with PVA and BN Additions

**DOI:** 10.3390/ma15051685

**Published:** 2022-02-24

**Authors:** Dilara Arıbuğa, Ufuk Akkaşoğlu, Buğra Çiçek, Özge Balcı-Çağıran

**Affiliations:** 1 Graduate School of Sciences and Engineering, Koç University, 34450 Istanbul, Turkey; daribuga15@ku.edu.tr; 2 Koç University Boron and Advanced Materials Application and Research Center, Koç University, 34450 Istanbul, Turkey; 3 Akcoat R&D Center, Ceramic Coatings Division, 2nd Industrial Zone, 54300 Sakarya, Turkey; ufuk.akkasoglu@akcoat.com; 4 Department of Metallurgical and Materials Engineering, Yıldız Technical University, 34210 Istanbul, Turkey; bcicek@yildiz.edu.tr; 5 Department of Chemistry, Koç University, 34450 Istanbul, Turkey

**Keywords:** glass-BN composites, PVA addition, low temperature sintering, microstructure, thermal conductivity, dielectric constant, LTCC

## Abstract

With the rapid development of the microelectronics industry, many efforts have been made to improve glass-ceramics’ sinterability, thermal conductivity, and dielectric properties, which are essential components of electronic materials. In this study, low-alkali borosilicate glass-ceramics with PVA addition and glass-BN composites were prepared and successfully sintered at 770 °C. The phase composition, density, microstructure, thermal conductivity, and dielectric constant were investigated. It was shown that PVA addition contributes to the densification process of glass-ceramics (~88% relative density, with closed/open pores in the microstructure) and improves the thermal conductivity of glass material from 1.489 to 2.453 W/K.m. On the other hand, increasing BN addition improves microstructures by decreasing porosities and thus increasing relative densities. A glass-12 wt. % BN composite sample exhibited almost full densification after sintering and presented apparent and open pores of 2.6 and 0.08%, respectively. A high thermal conductivity value of 3.955 W/K.m and a low dielectric constant of 3.00 (at 5 MHz) were observed in this material. Overall, the resulting glass-ceramic samples showed dielectric constants in the range of 2.40–4.43, providing a potential candidate for various electronic applications.

## 1. Introduction

With the rapid development of technology, electronic devices are becoming smaller and more multidirectional. Thus, the miniaturization and integration of electronic devices are receiving increasing attention [1,2,3,4,5,6,7,8,9]. Various glasses and glass-ceramics are widely used or considered for use as components of electronic materials due to their promising properties [10,11,12,13,14]. Porosity, hardness, strength, translucency, opacity, thermal expansion, temperature stability, chemical durability, thermal conductivity, dielectric loss, and resistivity can be designed and tailored in glass-ceramics [2,3,10,13]. Among glass-ceramic materials, low-temperature co-fired ceramics (LTCC) are multilayered ceramic materials that are important due to their exceptional physical and chemical properties [7,10,15,16]. LTCC technology is widespread in automobile and mobile technology, even in the military. It attracts attention for wireless communication, antenna, telescope mirror blanks, computer applications, chip modules, and biomedical sensors [4,7,10,11,15,17,18]. The significant characteristics of LTCC are excellent dielectric properties, high thermal conductivity, cost-effectiveness, and sintering temperature below the melting points of metal electrodes (Ag or Cu or Au). Thus, sintering below 950 °C makes it unique for miniaturized and integrated electronic devices [1,3,4,7,8,11,19,20,21,22]. Besides, glass content embedded in a ceramic system provides multifunctional characteristics, such as high strength and high durability [10,14,23]. In the area of ceramics, many materials show enough dielectric performance for electronic devices. However, these applications have high sintering temperatures and some conductivity problems, and are not cost-efficient [10,11]. Therefore, the quest for further investigation in the field of LTCC materials with unique properties, such as low dielectric constant, high thermal conductivity, cost-effectiveness, and low-sintering temperature is essential [2,3,11].

Many glass-ceramics are commercially produced, including MgO-Al_2_O_3_-SiO_2_, MgO-B_2_O_3_-SiO_2_, Li_2_O-Al_2_O_3_-SiO_2_, and CaO-B_2_O_3_-SiO_2_ systems [24,25,26,27,28,29,30,31]. In addition to these, boron oxides are potential candidates (e.g., borosilicate glasses) for glass-ceramics due to their superior characteristics, such as low melting temperature, low thermal expansion coefficient, high resistance, and surface tension [13,31,32]. The sintering additives are used to increase densification and thermal conductivity value. Therefore, the selection of additives is as crucial as the selection of glass-ceramic powders. In previous studies, hBN, AlN, Si_3_N_4_, and Al_2_O_3_ were used as ceramic additives or fillers [13,33,34,35]. Within the selection process, hexagonal boron nitride (hBN) is used to increase thermal conductivity [36,37]. Using boron oxides such as boron trioxide with alumina composites is the new approach for many glass applications, including LTCC [32,38]. These materials are synthesized in two steps: glass production and sintering. However, methods vary. They include such as solid-state, melt quenching, water-quenching, non-isothermal, and sol-gel [3,4,26,27,39].

Several ongoing studies focus on LTCC materials with different systems and additives. For example, J. Liu et al. studied the influence of B_2_O_3_ addition instead of Y_2_O_3_ in B_2_O_3_-Al_2_O_3_-SiO_2_ glass-ceramic systems to investigate differences in structure and properties and observed that B_2_O_3_ addition led to an increase in bending strength [40]. Similarly, Z. Qing et al. examined the Li_2_O-MgO-Al_2_O_3_-SiO_2_ glass-ceramics synthesized by the non-isothermal method, discussed adding different additives such as zirconia, and investigated the bending strength, crystallization, bulk density, and shrinkage of glass ceramics [4]. In addition, M. K. Zitani et al. reported CaO-MgO-SiO_2_ diopside-based glass-ceramics obtained by the melt quenching technique/two-step sintering and investigated crystallization behavior, sinterability, thermal behavior, relative density, degree of crystallization, and dielectric properties [3]. However, few studies have used PVA and BN comparatively as additives to show their potential effects on sintering process, as well as the thermal and dielectric properties of glass-ceramics. 

In the present study, glass-ceramic materials were investigated using borosilicate glass particles consisting of oxides such as SiO_2,_ B_2_O_3_, Al_2_O_3_, and alkali oxides. As-prepared glass particles were mixed with the additives and sintered below 800 °C. Low-alkali borosilicate glass was chosen due to its unique characteristics of low dielectric constant, high mechanical strength, thermal properties, and chemical stability [40]. The presence of Al_2_O_3_ and SiO_2_ improves the mechanical and chemical properties; however, deterioration can be seen at higher temperatures [40]. To prevent this problem, B_2_O_3_ was used in the composition. The low-alkali borosilicate glass powders with and without PVA addition and borosilicate glass–BN composite materials were examined in this paper to reveal the effects of additives on the microstructure, sinterability, thermal conductivity, and dielectric properties of the final ceramics. Finally, the features and prospects of the examined glass-ceramic materials in electronic systems, where low-temperature sintering, high thermal conductivity, and low dielectric constant are required, are discussed. 

## 2. Experimental Method

### 2.1. Preparation of Powders and Sintered Ceramics

The glass with a chemical composition of SiO_2_ (71 wt. %.), B_2_O_3_ (26 wt. %), Al_2_O_3_ (1 wt. %), and alkali metal oxides (0.5 wt. % of Li_2_O, 0.5 wt. % of Na_2_O, and 1 wt. % of K_2_O) was prepared through conventional melt quenching method. High-purity (>99%) starting materials were mixed and melted at 1500 °C for 1 h. The subsequent glass melt was quenched by water to obtain frits. The glass preparation method details are given in [41]. The obtained glass was milled and sieved to reach a particle size of <20 µm. The obtained glass powders were mixed with hexagonal boron nitride (hBN) at 3, 6, 9, and 12 wt. % by high-energy ball milling. Milling experiments were performed using a Spex 8000D Mixer Mill (SPEX SamplePrep, Metuchen, NJ, USA) equipped with hardened steel balls and vials. The powders were milled at room temperature for 30 min using a ball-to-powder mass ratio (BPR) of 10:1 and at a milling speed of 1200 rpm. After milling, glass composites were uniaxially pressed at 300 MPa to form pellets with a diameter of 10 mm. The pellets were sintered at 720 and 770 °C for 1 h in a tube furnace and then naturally cooled. Sintering temperatures were determined according to the heating microscope analysis results of the glass powders. For the purpose of comparison, the pressing and sintering of glass powders were studied in identical conditions with and without polyvinyl alcohol (PVA) binder at the amounts of 1 and 3 wt. %. The glass-ceramics with and without PVA and glass composites with hBN sintered at 720 and 770 °C are hereafter termed G3BN@770, G6BN@770, G9BN@770, G12BN@770, G1PVA@770, G3PVA@770, G@770, G@720, G1PVA@720, G3BN@720, and G6BN@720. Table 1 summarizes the sample names and their production conditions. 

### 2.2. Characterizations

Rigaku Miniflex X-ray diffractometer (XRD, CuK_α_) and International Center for Diffraction Data (ICDD) database were used to analyze the phase structure of the samples. To obtain a scratch-free mirror finish for microstructural analyses, samples were subjected to a typical metallographic preparation procedure. Specimens were cold-mounted by using epoxy resin and ground and polished in a Struers™ Tegrapol-15 instrument. Zeiss Ultra Plus field emission scanning electron microscope (FE-SEM) equipped with energy-dispersive X-ray spectrometer (EDS) was used for microstructural and morphological investigation of the polished samples. A secondary electron (SE) detector was used to obtain the SEM images with an acceleration voltage of 8 kV and working distance of 8 mm. Bruker XFlash 5010 EDS detector (Bruker, Billerica, MA, USA) with 123 eV resolution was used for EDS measurements and EDX mapping. The quality of the samples was also examined by using an optical microscope (SOIF). Average density values of sintered products were determined in ethanol using the Archimedes method by taking three iterative measurements for each sample. Relative Archimedes density (%) was found by dividing average experimental density (obtained by Archimedes method) by the bulk density of the glass-ceramic (2.02 g/cm^3^) [41] produced and multiplied by 100. Next, apparent porosity was found by Equation (1), where the average density was the measured experimental density obtained via the Archimedes method and dimensional density was the density calculated by dividing the mass of the prepared samples by their volume.

(1) $$\mathrm{apparent}\;\mathrm{porosity}=\left(1-\frac{\mathrm{dimensional}\;\mathrm{density}}{\mathrm{average}\;\mathrm{density}}\;\right)\times 100$$

To find open porosity (%), mass in the air of the sample is subtracted from the mass in the pure water and multiplied by 100. The thermal diffusivity was measured at room temperature on the disc-shaped cylindrical pellets. The thermal diffusivity measurements, D, were carried out using a NETZSCH LFA HT467 (NETZSCH-Gerätebau GmbH, Selb, Germany) laser flash apparatus. The thermal conductivity of the samples was calculated using the relation: *κ = DC_P_d*, where C_P_ is the theoretical heat capacity of SiO_2_ and d is the experimental density. The dielectric properties at 5 MHz were investigated by using a Hioki IM3570 (HIOKI E.E. Corporation, Nagano, Japan) impedance analyzer according to the parallel plate method. The parallel surfaces of the samples were coated with conductive gold-palladium alloy by using a quorum sputter coater. A sinusoidal voltage was applied to create alternating electric fields and thus polarize the sample. The dielectric constant was calculated from capacitance measurement at room temperature according to Equation (2), where *K* is the dielectric constant, C is the capacitance, d is the thickness of the sample, Ɛ is the permittivity of vacuum, and A is the area of the sample.

(2) $$K=\frac{C.d}{\varepsilon .A}$$

## 3. Results and Discussion

**Figure 1:**
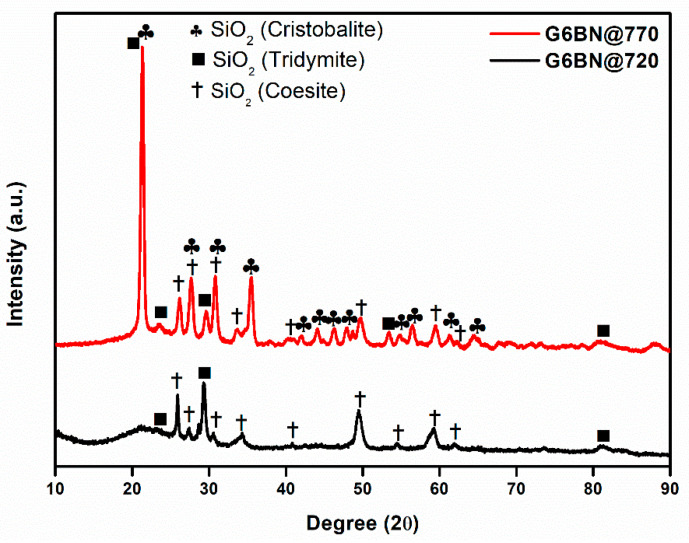
XRD patterns of glass ceramics with 6 wt. % hBN sintered at 720 and 770 °C for 1 h.

**Figure 2:**
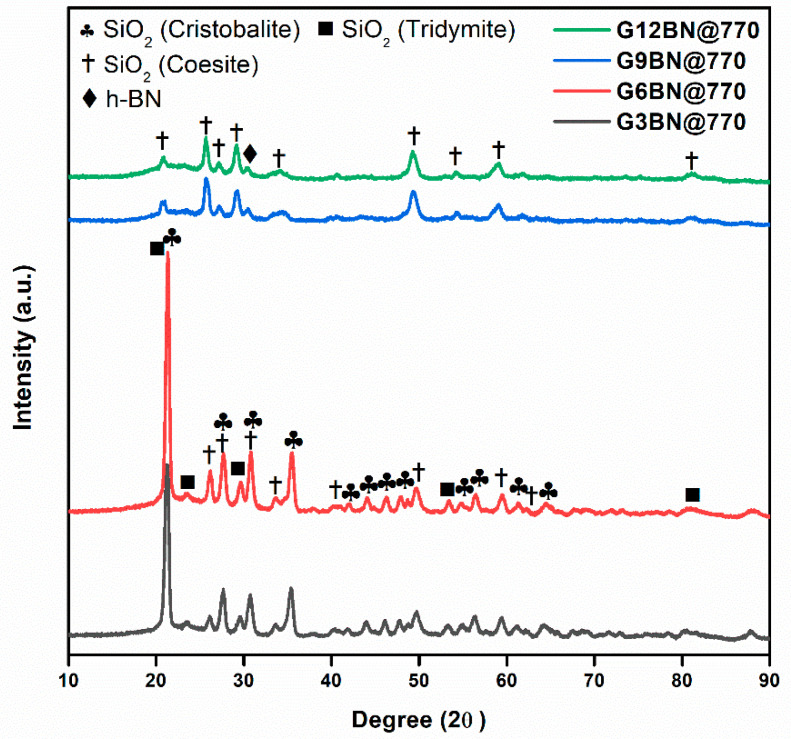
XRD patterns of glass ceramics with 3, 6, 9, and 12 wt. % hBN sintered at 770 °C for 1 h.

**Figure 3:**
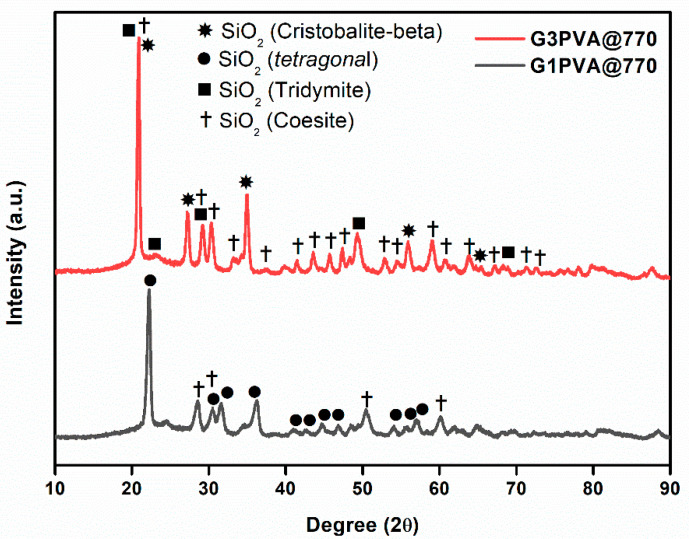
XRD patterns of glass ceramics with 1 and 3 wt. % PVA sintered at 770 °C for 1 h.

All glass-ceramics sintered at 720 and 770 °C were analyzed by the XRD technique, which are presented in Figure 1, Figure 2 and Figure 3. Table S1 (in the supporting information file) shows all the obtained phases with their corresponding ICDD Card numbers and crystal structures. To see the effect of sintering temperature, Figure 1 representatively shows the XRD patterns of glass ceramics with BN additive that sintered at different temperatures, 720 and 770 °C, for 1 h. It is clear from Figure 1 that the crystallinity of the glass-ceramics with BN additive increased at 770 °C in comparison to 720 °C. Similarly, it was also observed in the XRD patterns of glass ceramics with PVA additives that more crystalline products were obtained at higher temperatures (not shown here). Furthermore, an increase in sintering temperature had an impact on crystallized SiO_2_ phase in the XRD patterns (Figure 1). Different crystallized phases occurred due to the temperature during sintering, so high content of SiO_2_ and B_2_O_3_ in the glass material induced more in situ cristobalite, tridymite, and coesite crystallization (Figure 1). On the other hand, BN phase could not be observed due to its lightness and the small amount in the overall sample (6 wt. % of BN). Figure 2 and Figure 3 show the XRD patterns of glass ceramics with BN and PVA additives that sintered at 770 °C for 1 h, indicating the difference between BN and PVA addition. Increasing the amount of BN in glass powder led to a decrease in crystallinity of the sintered glass-ceramics in general. G3BN@770 and G6BN@770 samples were present the formation of cristobalite, coesite, and tridymite. On the other hand, the G9BN@770 and G12BN@770 samples were more amorphous than the others and showed only in situ coesite crystallization (Figure 2). In addition, h-BN phase was clearly seen in these samples due to the high amount of BN in the overall sample (9 and 12 wt. % BN). The presence of BN phase indicates that BN did not react with the glass phase during sintering to form undesired products. As seen in Figure 3, the XRD patterns of the G1PVA@770 and G3PVA@770 samples also gave different crystal structures of SiO_2_. Increasing the PVA content from 1 to 3 wt. % resulted in the formation of a considerable amount of different crystal forms of SiO_2_ phases, such as cristobalite-beta and tridymite (Figure 3). In addition, a small peak shift was observed in the XRD pattern of the G3PVA@770 sample, most likely due to an incorporated impurity phase derived from PVA additive. This shows the effects of PVA addition on the phase composition of the glass ceramics, where, possibly, a reaction and subsequent crystallization occurred between the components during sintering. 

In order to draw a comparison, different density and porosity measurements/calculations were performed, as shown in Table 2. As mentioned in the experimental procedure, the open porosity calculation results differed from the apparent porosity results. As shown in Table 2, the glass-BN composites sintered at 720 °C exhibited lower density and higher porosity values than those sintered at 770 °C. By contrast, the glass-ceramics sintered at 720 °C with and without PVA addition produced better density and porosity results than those sintered at 770 °C. This indicates that BN addition slightly increases the sintering temperature of glass ceramics and provides nearly complete (85.64%) and full densification (100%) at 770 °C, as 9 and 12 wt. % amounts are used, respectively. The reason for the 103.96% relative density is related to the higher bulk density of BN than that of the reference glass material (2.02 g/cm^3^). Furthermore, in BN-glass composites sintered at 770 °C, there was a similar trend between the apparent and open porosity results: both were decreased by increasing the BN content. On the other hand, concerning the PVA content at the different sintering temperatures, no comparable trend could be observed between the density and porosity of the samples. PVA addition slightly contributed to the densification process of the glass-ceramics sintered at 770 °C, and the G1PVA@770 and G3PVA@770 samples exhibited a relative density of 87.62%. Based on the density and porosity calculations, G12BN@770 had the highest relative density (100%) and lowest apparent porosity (2.6%) values among the samples, which means that increasing the amount of BN significantly enhanced the densification and decreased the porosity. 

**Figure 4:**
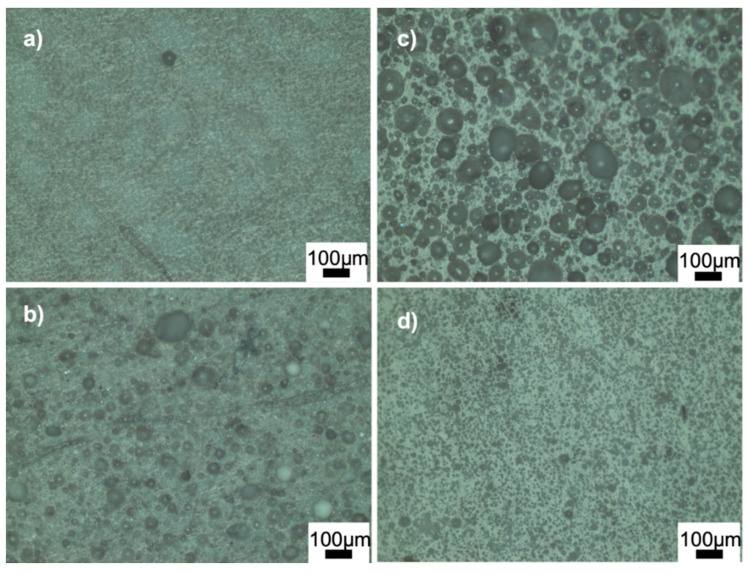
Optical microscope images of the ceramics sintered at 720 °C: (**a**) G@720, (**b**) G1PVA@720, (**c**) G3BN@720, (**d**) G6BN@720.

**Figure 5:**
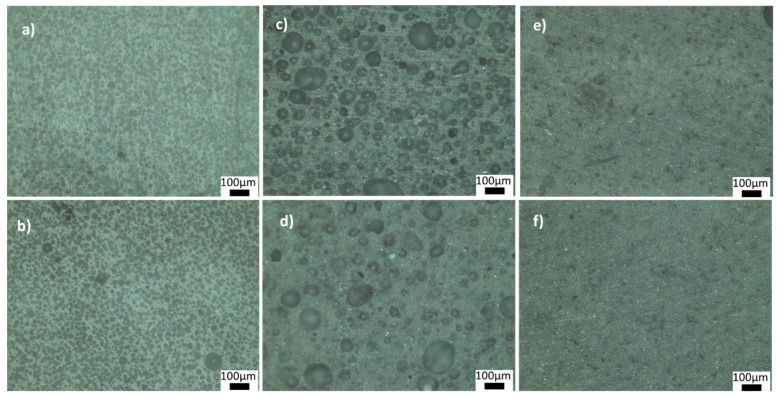
Optical microscope images of the ceramics sintered at 770 °C: (**a**) G1PVA@770, (**b**) G3PVA@770, (**c**) G3BN@770, (**d**) G6BN@770, (**e**) G9BN@770, (**f**) G12BN@770.

Figure 4 and Figure 5 show the optical microscope images of glass-ceramics sintered at 720 and 770 °C, respectively. The increase in the amount of PVA led to an increase in the number of closed pores, shown in Figure 4a,b. By contrast, the number of large, closed pores reduced when the amount of BN increased at 720 °C (Figure 4d). These results showed that the density estimates were consistent with the optical microscope images. As shown in Figure 5a,b, PVA had a positive effect on reducing the large closed pores; however, there were still small closed pores throughout the sample. As shown in Figure 5c,d, closed porosity was observed in the microstructures of the G3BN@770 and G6BN@770 samples. On the other hand, the microstructures of the G9BN@770 and G12BN@770 samples did not show any porosity (Figure 5e,f), which is also comparable to the high density and low porosity values in Table 2. This shows that the addition of 9 and 12 wt. % BN reduced not only the apparent and open porosity but also the closed pores in the microstructure. It has already been reported that a large number of closed pores is harmful to the thermal conductivity of the material and, hence, the performance of sintered products [42]. 

**Figure 6:**
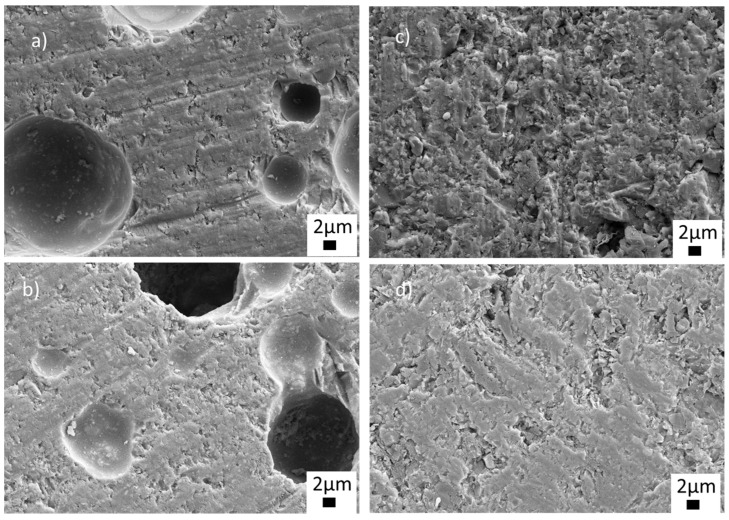
SEM images of the sintered glass-ceramics at 5.00 KX (**a**) G1PVA@770, (**b**) G3PVA770, (**c**) G9BN@770, (**d**) G12BN@770.

To observe the microstructures in detail, FE-SEM analyses were carried out at different magnifications on the samples sintered at 770 °C. Figure 6 shows the low-magnification SEM images of the sintered ceramics with BN and PVA additions. As seen from Figure 6a,b, closed and open (sizes up to 20 µm) pores were visible in the microstructures of the glass-ceramics sintered with PVA addition. The structure, size and distribution of the pores can be seen more clearly in Figure S1 in the supporting information file (SI). Closed and open pores are marked by red and blue arrows, respectively (Figure S1, SI). On the other hand, it was observed that BN addition eliminated both closed and open pores, as shown in the low magnification images in Figure 6c,d. Thus, the SEM images in Figure 6c,d exhibited near-full and full densification without any visible pores, indicating an enhancement of the sinterability of the glass-ceramic material. The glass-BN composite sample containing the highest BN amount showed a uniform, dense microstructure, as seen in Figure 6d. 

**Figure 7:**
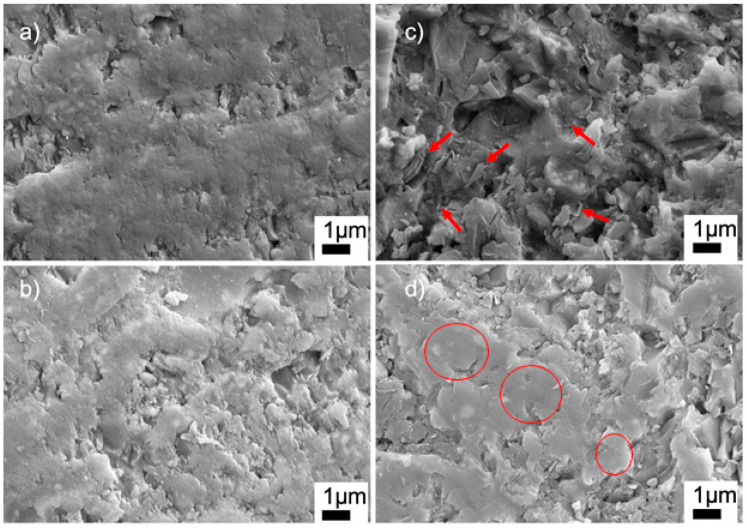
SEM images of sintered glass-ceramics at 20 KX (**a**) G1PVA@770, (**b**) G3PVA@770, (**c**) G9BN@770, (**d**) G12BN@770.

Figure 7 shows the high-magnification SEM images of the sintered ceramics with BN and PVA additions. The SEM image of the G9BN@770 sample shows the nanoplates of BN, which were randomly agglomerated in some regions of the sample, as marked by the arrows in Figure 7c. However, the G12BN@770 sample had BN particles with mostly dispersed and aligned structures, as shown by the circles in Figure 7d. This structure could inhibit the pore formation and, correspondingly, enhance densification, as supported by the density measurements in Table 2. A similar dispersed structure can also be seen in the high-magnification SEM images of the G1PVA@770 and G3PVA@770 samples (Figure 7a,b). However, these regions do not represent the whole sample, where micron-scale closed pores are visible throughout the microstructure. The SEM images at lower magnifications of these samples can be seen in Figure S1 in the supporting information file (SI). EDS area analyses of the samples are given in Figures S2–S5 (SI). All measurements gave the elements of Si, Al, B, and O, as expected. The EDS measurement carried out on the G9BN@770 and G12BN@770 samples additionally showed the presence of N element and high-intensity B peaks, indicating the existence of BN phase in the structure. These results are consistent with the XRD patterns in Figure 2, where the BN phase was observed in the G9BN@770 and G12BN@770 samples. 

**Figure 8:**
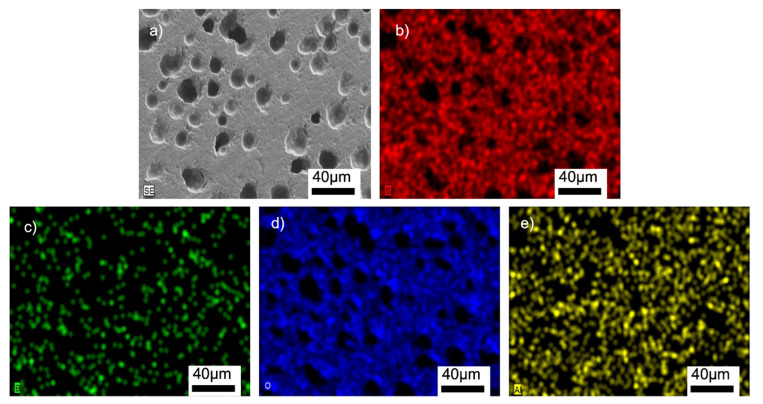
SEM/EDX analyses of the G3PVA@770 sample: (**a**) SEM image, (**b**) EDX mapping of Si, (**c**) EDX mapping of B, (**d**) EDX mapping of O, (**e**) EDX mapping of Al.

**Figure 9:**
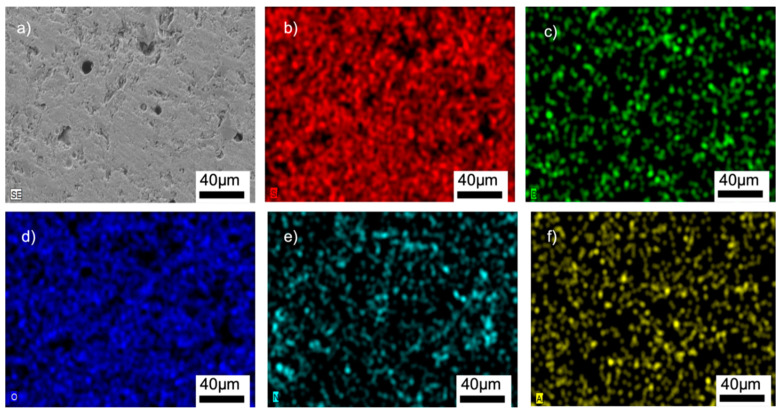
SEM/EDX analyses of the G12BN@770 sample: (**a**) SEM image, (**b**) EDX mapping of Si, (**c**) EDX mapping of B, (**d**) EDX mapping of O, (**e**) EDX mapping of N, (**f**) EDX mapping of Al.

Figure 8, Figure 9, Figures S6 and S7 (SI) show the SEM/EDX analyses of the samples. The homogeneous distributions of the Si, B, O, and Al elements can be clearly seen in all the microstructures. The glass-BN composites additionally gave the signals of N element, which was also uniformly distributed. The EDX analyses suggested that the main problems encountered in general glass-ceramic processes, such as a lack of well-distributed particles, the occurrence of clustered areas, or the formation of secondary phases, were not observed in the prepared samples. A large amount of Si and O was observed in the EDX analyses, suggesting the absence of any reaction between SiO_2_ and other additional elements. This might be related to the used preparation process of the composites containing solid-state mechanical milling at room temperature, followed by low-temperature sintering. 

Table 3 shows the measured thermal diffusivity values with corresponding standard deviations and calculated thermal conductivity values of the samples. For the calculation of thermal conductivity, the theoretical heat capacity was taken as 1.245 j/g.K, and density was taken as the experimental Archimedes density values in Table 2. As shown in Table 3, both glass-ceramics and composites sintered at 720 °C displayed lower values than those sintered at 770 °C. When a small amount of BN was added to the composite, the thermal conductivity of the glass-ceramic increased from 1.489 to 1.729 W/K.m. As the BN content increased from 3 to 12% wt., the thermal conductivity improved from 1.729 to 3.955 W/K.m. Interestingly, the glass-ceramics sintered at 770 °C with PVA addition also presented comparably high thermal conductivity values (2.107 and 2.453 W/K.m), which were greater than that of the glass-ceramic itself (1.489 W/K.m). Despite the presence of closed pores in the microstructures of the G1PVA@770 and G3PVA@770 samples, enhanced thermal conductivities were obtained. This was most likely related to the structure of the pores (which were smaller in size and distributed uniformly) and the excellent densification process at 770 °C, indicating the importance of the selected sintering temperature. The relationship between pore size/distribution/amount and thermal conductivity has been previously reported for silica ceramics [43,44]. The experimental and theoretical research results showed that pore size, especially, is a decisive influencing factor in the thermal properties products [43,44,45]. On the other hand, the highest value, of 3.955 W/K.m, was obtained for the G12BN@770 sample, which is consistent with the reported high density, low porosity, and improved microstructure of the sample. Furthermore, the high thermal conductivity of BN phase contributed to this, since no deterioration of BN was observed, thanks to the utilized sintering temperature and preparation processes. In previous studies, AlN, Si_3_N_4_, and Al_2_O_3_ were also used instead of BN [13,33,34,35]. However, it was shown that the use of glass-ceramics with BN is a more effective way to contribute to composites than AlN by comparing their thermal conductivity values with different glass-ceramic content [33]. 

Table 4 shows the dielectric properties of the samples sintered at 770 °C. It was observed that the dielectric constant of the glass itself (G@770) was relatively low and dielectric loss was relatively high in comparison to the reference work [41]. This was attributed to the closed porosities that were present in the microstructure, since microstructural flaws increase dielectric loss and the decrease dielectric constant [46,47]. This is also consistent with the decreased dielectric loss of the glass-BN composites with improved densities, where the dielectric loss decreased from ~0.11 to 0.04 for G3BN@770 and G12BN@770, respectively. As shown in Table 4, the dielectric constants were calculated to be between 2.40 and 4.43 at 5 MHz. With the increase in the BN content to 6 wt. %, the dielectric constant of the samples increased from 2.42 to 4.43, and BN content above 6 wt. % led to a decrease in the values. In addition, the sample with 1 wt. % PVA addition exhibited a dielectric constant of 2.40, whereas the G3PVA@770 sample could not be measured due to the deterioration of its shape. Considering all the results, optimum values were achieved for the G12BN@770 sample, which had full densification (Table 2), a high thermal conductivity, of 3.955 W/K.m (Table 3), and a low dielectric constant, of 3.00 (Table 4). Therefore, it can be inferred that the hBN phase itself effectively optimized both microstructure and density, as well as dielectric and thermal properties. Thus, the G12BN@770 sample has comparable dielectric and thermal properties to similar products reported in previous studies, with a densification temperature of 770 °C [48]. On the other hand, the highest dielectric constant, of 4.43, was obtained for the G6BN@770. The obtained dielectric constants of the glass-ceramics were also comparable to the results of the bulk glass sample itself (4.07–4.50 at 1 MHz) [41]. The obtained results were compared to commercially produced LTCC glass-ceramics sintered at 800–900 °C, which also used B_2_O_3_ to lower the sintering temperature. It was observed that they had comparable thermal conductivity values (2.0–4.9W/m.K) to those in the present study [26,48,49]. Other commercial products with similar glass-ceramic compositions have dielectric constants between ~ 4 and 5 [50,51]. It is worth mentioning here that the glass-ceramics were sintered at a lower temperature than commercially produced products with similar thermal and dielectric properties. In addition, the addition of BN and PVA had an apparent impact on the thermal and dielectric properties of the obtained glass-ceramics [48]. The results indicate that borosilicate glass-ceramics with additives can be potential candidates for LTCC applications. 

## 4. Conclusions

In this study, glass-ceramic materials were prepared based on borosilicate glass and glass/PVA ceramics and glass-hBN composites by mechanical milling and solid-phase sintering methods. Utilizing the glass composition and the used additions, the sintering temperature decreased to 770 °C, and near-fully or fully densified samples were obtained. PVA addition contributed to the densification process of the glass-ceramics (~88% relative density, with closed/open pores in the microstructure) and led to a thermal conductivity value of 2.453 W/K.m for the glass-3 wt.% PVA sample sintered at 770 °C. The thermal conductivity values were calculated in the range between 0.950 and 3.955 W/K.m, whereas the dielectric constant values were obtained between 2.40 and 4.43 for all the samples. A thermal conductivity of 3.955 W/K.m and a dielectric constant of 3.00 (at 5 MHz) were achieved for the glass-12 wt.% hBN composite sample sintered at 770 °C, which exhibited full densification, improved microstructure, and apparent and open pores of 2.6 and 0.08%, respectively. The improved microstructure, good sinterability, high thermal conductivity, and low dielectric constant make the prepared borosilicate glass-ceramics with PVA addition and borosilicate glass-BN composites promising candidates for microelectronic devices. In addition, the samples’ low sintering temperature, of 770 °C, provides a new potential for LTCC applications. 

## Figures and Tables

**Table 1:** Sample names and their production conditions.

Sample Name	% of Glass Powder	% of BN	% of PVA	Sintering Temperature (°C)
G3BN@770	97	3	-	770
G6BN@770	94	6	-	770
G9BN@770	91	9	-	770
G12BN@770	88	12	-	770
G1PVA@770	99	-	1	770
G3PVA@770	97	-	3	770
G@770	100	-	-	770
G@720	100	-	-	720
G1PVA@720	99	-	1	720
G3BN@720	97	3	-	720
G6BN@720	94	6	-	720

**Table 2:** Density and porosity results of the glass-ceramics.

Sample Name	Average Archimedes Density (g/cm^3^)	Relative Archimedes Density (%)	Apparent Porosity (%)	Open Porosity (%)
G3BN@770	1.508 ± 0.002	74.75	13.5	0.91
G6BN@770	1.524 ± 0.002	75.25	11.9	0.25
G9BN@770	1.733 ± 0.001	85.64	9.0	0.14
G12BN@770	2.100 ± 0.002	103.96	2.6	0.08
G1PVA@770	1.773 ± 0.000	87.62	12.2	0.04
G3PVA@770	1.770 ± 0.003	87.62	9.5	0.13
G@770	1.769 ± 0.002	87.12	13.7	0.11
G@720	1.938 ± 0.005	96.04	10.7	0.07
G1PVA@720	1.904 ± 0.001	94.06	14.6	0.51
G3BN@720	1.402 ± 0.010	69.30	8.98	1.27
G6BN@720	1.662 ± 0.002	82.18	13.53	0.99

**Table 3:** Thermal conductivity of the samples.

Sample Name	Diffusivity (mm^2^/s)	Standard Deviation (mm^2^/s)	Thermal Conductivity (W/K.m)
G3BN@770	0.911	0.012	1.729 ± 0.02
G6BN@770	1.395	0.006	2.620 ± 0.01
G9BN@770	1.191	0.005	2.570 ± 0.01
G12BN@770	1.466	0.002	3.955 ± 0.01
G1PVA@770	0.954	0.022	2.107 ± 0.05
G3PVA@770	1.113	0	2.453 ± 0.00
G@770	0.676	0.004	1.489 ± 0.01
G@720	0.670	0.007	1.617 ± 0.02
G1PVA@720	0.591	0.006	1.401 ± 0.01
G3BN@720	0.785	0.003	1.371 ± 0.01
G6BN@720	0.459	0.005	0.950 ± 0.01

**Table 4:** Dielectric properties of the samples.

Sample Name	Dielectric Constant (@ 5 MHz)	Dielectric Loss (@ 5 MHz)
G@770	2.42 ± 0.1	0.2025 ± 0.01
G3BN@770	3.84 ± 0.1	0.1068 ± 0.01
G6BN@770	4.43 ± 0.1	0.0373 ± 0.01
G9BN@770	2.84 ± 0.1	0.0272 ± 0.01
G12BN@770	3.00 ± 0.1	0.0388 ± 0.01
G1PVA@770	2.40 ± 0.1	0.0296 ± 0.01
G3PVA@770	N/A	N/A

## Data Availability

The data presented in this study are available on request from the corresponding author. The data are not publicly available due to their association with an ongoing study.

## Supplementary Materials

The following supporting information can be downloaded at: https://www.mdpi.com/article/10.3390/ma15051685/s1, Figure S1: SEM images of sintered glass ceramics at 1.00 KX (a) G1PVA@770, (b) G3PVA770; Figure S2. EDS analysis of the G1PVA@770 sample; Figure S3. EDS analysis of the G3PVA@770 sample; Figure S4. EDS analysis of the G9BN@770 sample; Figure S5. EDS analysis of the G12BN@770 sample; Figure S6. SEM/EDX analyses of the G1PVA@770 sample (a) SEM image, (b) EDX mapping of Si, (c) EDX mapping of B, (d) EDX mapping of O, (e) EDX mapping of Al.; Figure S7. SEM/EDX analyses of the G9BN@770 sample (a) SEM image, (b) EDX mapping of Si, (c) EDX mapping of B, (d) EDX mapping of O, (e) EDX mapping of Al.; Table S1. ICDD Card numbers and crystal structures for the obtained phases.

## References

[1] Zhang P., Sun K., Wu S., Xiao M. (2019). Microwave dielectric properties of low temperature co-fired ceramics LiMg_1−x_A_x_PO_4_ (A = Mn, Ca, 0.02 ≤ x ≤ 0.08). Mater. Lett..

[2] Yu Y., Hao X., Song L., Li Z., Song L. (2016). Synthesis and characterization of single phase and low temperature co-fired cordierite glass-ceramics from perlite. J. Non-Cryst. Solids.

[3] Zitani M.K., Ebadzadeh T., Banijamali S., Riahifar R., Rüssel C., Abkenar S.K., Ren H. (2018). High quality factor microwave dielectric diopside glass-ceramics for the low temperature co-fired ceramic (LTCC) applications. J. Non-Cryst. Solids.

[4] Qing Z., Li B. (2018). Non-isothermal crystallization kinetics and properties of low temperature co-fired LiAlSi_3_O_8_-based glass-ceramics using zirconia additive. J. Non-Cryst. Solids.

[5] Li H., Huang Z., Cheng L., Kong S., Liu S. (2017). Structure and dielectric properties of novel low temperature co-fired Bi_2_O_3_-RE_2_O_3_-MoO_3_ (RE = Pr, Nd, Sm, and Yb) based microwave ceramics. Ceram. Int..

[6] Zhang Z., Tang Y., Li J., Chen J., Yang A., Wang Y., Zhai Y., Ao L., Su C., Xing X. (2020). High-Q and near-zero τ composite Li_2_Mg_2_TiO_5_-Sr_3_(VO_4_)_2_ ceramics for low-temperature co-fired ceramic applications. Ceram. Int..

[7] Tao B., Wang W., Liu H., Du T., Wu H., Xing C., Wang D., Zhang Y. (2021). Low-temperature sintering LiF-doped Li_4_Mg_3_[Ti_0_._6_(Mg_1/3_Nb_2/3_)_0.4_]_2_O_9_ microwave dielectric ceramics for LTCC applications. Ceram. Int..

[8] Wu J., Li Z., Huang Y., Li F., Yang Q. (2014). Fabrication and characterization of low temperature co-fired cordierite glass–ceramics from potassium feldspar. J. Alloy. Compd..

[9] George S., Sebastian M.T. (2009). Effect of lithium-based glass addition on the microwave dielectric properties of Ca[(Li_1/3_Nb_2/3_)_1−x_Ti_x_]O_3−δ_ ceramics for LTCC applications. J. Alloy. Compd..

[10] Bermejo R., Supancic P., Krautgasser C., Morrell R., Danzer R. (2013). Subcritical crack growth in Low Temperature Co-fired Ceramics under biaxial loading. Eng. Fract. Mech..

[11] Cui X., Zhou J. (2008). A simple and an effective method for the fabrication of densified glass-ceramics of low temperature co-fired ceramics. Mater. Res. Bull..

[12] Fernandes J., Barcelona P., Blanes M., Padilla J., Ramos F., Cirera A., Xuriguera E. (2021). Study of mixing process of low temperature co-fired ceramics photocurable suspension for digital light processing stereolithography. Ceram. Int..

[13] Yuan L., Liu B., Shen N., Zhai T., Yang D. (2014). Synthesis and properties of borosilicate/AlN composite for low temperature co-fired ceramics application. J. Alloy. Compd..

[14] Bermejo R., Supancic P., Kraleva I., Morrell R., Danzer R. (2011). Strength reliability of 3D low temperature co-fired multilayer ceramics under biaxial loading. J. Eur. Ceram. Soc..

[15] Jędrychowska A., Malecha K., Cabaj J., Sołoducho J. (2015). Laccase biosensor based on low temperature co-fired ceramics for the permanent monitoring of water solutions. Electrochim. Acta.

[16] Malecha K., Maeder T., Jacq C., Ryser P. (2011). Structuration of the low temperature co-fired ceramics (LTCC) using novel sacrificial graphite paste with PVA–propylene glycol–glycerol–water vehicle. Microelectron. Reliab..

[17] Huang C.-L., Chu T.-M., Tsai M.-H. (2021). A low-loss, low temperature sintering dielectric using Ba_1_-SrMg_2_(VO_4_)_2_ ceramics and its applications at microwave frequencies. Mater. Sci. Eng. B.

[18] Baeza M., López C., Alonso J., López-Santín J., Alvaro G. (2010). Ceramic Microsystem Incorporating a Microreactor with Immobilized Biocatalyst for Enzymatic Spectrophotometric Assays. Anal. Chem..

[19] Zhou H., Wang K., Sun W., Chen X., Ruan H. (2018). Phase composition, singtering behavior and microwave dielectric properties of M_2_BiLi_2_V_3_O_12_ (M = Zn, Ca) low temperature co-fired ceramics. Mater. Lett..

[20] Li J., Su H., Sun Y., Wang G., Gao F., Han X., Liang Z., Li Q. (2021). Enhancement of structural and microwave properties of Zn^2+^ ion-substituted Li_2_MgSiO_4_ ceramics for LTCC applications. Ceram. Int..

[21] Zhang Q.-L., Zou D., Yang H. (2011). Microwave dielectric properties of Ba_3_Ti_4−x_(Zn_1_/_3_Nb_2_/_3_)_x_Nb_4_O_21_ for low temperature co-fired ceramics. J. Eur. Ceram. Soc..

[22] Shigeno K., Li M., Fujimori H. (2021). Development of novel temperature-stable Al_2_O_3_–TiO_2_-based dielectric ceramics featuring superior thermal conductivity for LTCC applications. J. Eur. Ceram. Soc..

[23] Krautgasser C., Danzer R., Deluca M., Supancic P., Aldrian F., Bermejo R. (2016). Subcritical crack growth in multilayer Low Temperature Co-fired Ceramics designed with surface compressive stresses. J. Eur. Ceram. Soc..

[24] Xia Y., Hu Y., Ren L., Luo X., Gong W., Zhou H. (2018). Manufacturing a high performance film of CaO-B_2_O_3_-SiO_2_ glass-ceramic powder with surface modification for LTCC application. J. Eur. Ceram. Soc..

[25] Kumari P., Tripathi P., Parkash O., Kumar D. (2016). Low Temperature Sintering and Characterization of MgO-B_2_O_3_-SiO_2_ Glass-Ceramics for LTCC Substrate Applications. Trans. Indian Ceram. Soc..

[26] Qing Z. (2018). The effects of B2O3 on the microstructure and properties of lithium aluminosilicate glass-ceramics for LTCC applications. Mater. Lett..

[27] Shao H., Wang T., Zhang Q. (2009). Preparation and properties of CaO–SiO_2_–B_2_O_3_ glass-ceramic at low temperature. J. Alloy. Compd..

[28] Chang C.-R., Jean J.-H. (1999). Crystallization Kinetics and Mechanism of Low-Dielectric, Low-Temperature, Cofirable CaO-B_2_O_3_-SiO_2_Glass-Ceramics. J. Am. Ceram. Soc..

[29] Chen G.-H. (2008). Sintering, crystallization, and properties of CaO doped cordierite-based glass–ceramics. J. Alloy. Compd..

[30] Kemethmuller S., Roosen A., Goetz-Neunhoeffer F., Neubauer J. (2006). Quantitative Analysis of Crystalline and Amorphous Phases in Glass–Ceramic Composites Like LTCC by the Rietveld Method. J. Am. Ceram. Soc..

[31] Hamzawy E., El-Kheshen A., Zawrah M. (2005). Densification and properties of glass/cordierite composites. Ceram. Int..

[32] Lima M., Monteiro R., Graça M., da Silva M.F. (2012). Structural, electrical and thermal properties of borosilicate glass–alumina composites. J. Alloy. Compd..

[33] Sun Z., Li W., Liu Y., Zhang H., Zhu D., Sun H., Hu C., Chen S. (2019). Design and preparation of a novel degradable low-temperature co-fired ceramic (LTCC) composites. Ceram. Int..

[34] Ma M., Liu Z., Li Y., Zeng Y., Yao D. (2013). Enhanced thermal conductivity of low-temperature sintered borosilicate glass–AlN composites with β-Si_3_N_4_ whiskers. J. Eur. Ceram. Soc..

[35] Luo X., Ren L., Xia Y., Hu Y., Gong W., Cai M., Zhou H. (2017). Microstructure, sinterability and properties of CaO-B_2_O_3_-SiO_2_glass/Al_2_O_3_ composites for LTCC application. Ceram. Int..

[36] Chiang T.H., Hsieh T.-E. (2006). A Study of Encapsulation Resin Containing Hexagonal Boron Nitride (hBN) as Inorganic Filler. J. Inorg. Organomet. Polym. Mater..

[37] Xu C., Li E., Zeng J., Wang Y., Wang T., Ge C., Zhang C., Wang Q., Gao T., Yao Y. (2021). Interfacial thermal conductance enhancement of BN/PVA composites via plasma activations of fillers. Compos. Commun..

[38] Hasanuzzaman M., Rafferty A., Sajjia M., Olabi A.-G., Hashmi S. (2016). Properties of Glass Materials. Reference Module in Materials Science and Materials Engineering.

[39] Guo Q., Li L., Yu S., Sun Z., Zheng H., Luo W. (2018). Temperature–stable dielectrics based on Ni–doped Bi_2_Zn_2_/_3_Nb_4_/_3_O_7_ pyrochlore ceramics for low temperature co-fired ceramic. J. Alloy. Compd..

[40] Liu J., Luo Z., Lin C., Han L., Gui H., Song J., Liu T., Lu A. (2019). Influence of Y_2_O_3_ substitution for B_2_O_3_ on the structure and properties of alkali-free B_2_O_3_-Al_2_O_3_-SiO_2_ glasses containing alkaline-earth metal oxides. Phys. B: Condens. Matter.

[41] Akkasoglu U., Sengul S., Arslan I., Ozturk B., Cicek B. (2021). Structural, thermal and dielectric properties of low-alkali borosilicate glasses for electronic applications. J. Mater. Sci. Mater. Electron..

[42] Feng X., Lv Y., Zhang L., Ding J., Sun J., Li X., Chen L., Zheng K., Zhang X., Tian X. (2020). High Performance of Low-Temperature-Cofired Ceramic with Al_2_O_3_/BN Biphasic Ceramics Based on B_2_O_3_–Bi_2_O_3_–SiO_2_–ZnO Glass. Adv. Eng. Mater..

[43] Sonnick S., Meier M., Ross-Jones J., Erlbeck L., Medina I., Nirschl H., Rädle M. (2019). Correlation of pore size distribution with thermal conductivity of precipitated silica and experimental determination of the coupling effect. Appl. Therm. Eng..

[44] Šveda M., Janík B., Pavlík V., Štefunková Z., Pavlendová G., Šín P., Sokolář R. (2016). Pore-size distribution effects on the thermal conductivity of the fired clay body from lightweight bricks. J. Build. Phys..

[45] Skibinski J., Cwieka K., Ibrahim S.H., Wejrzanowski T. (2019). Influence of Pore Size Variation on Thermal Conductivity of Open-Porous Foams. Mater..

[46] Yamamoto J.K., Kata K., Shimada Y. (1989). Fabrication of controlled porosity in a tape cast glass ceramic substrate material. Mater. Lett..

[47] Kata K., Shimada Y. (1992). Low Dielectric Constant Glass-Ceramic Composite Controled Isolated Porosity. J. Ceram. Soc. Jpn..

[48] Bilaç O., Duran C. (2021). Al_2_O_3_/glass/hBN composites with high thermal conductivity and low dielectric constant for low temperature cofired ceramic applications. J. Asian Ceram. Soc..

[49] Sebastian M.T. (2008). Dielectric Materials for Wireless Communication.

[50] Zhou J. (2012). Towards rational design of low-temperature co-fired ceramic (LTCC) materials. J. Adv. Ceram..

[51] Sebastian M.T., Ubic R., Jantunen H. (2017). Microwave Materials and Applications, 2 Volume Set.

