# Supplementary material for: Enhanced Sinterability, Thermal Conductivity and Dielectric Constant of Glass-Ceramics with PVA and BN Additions
Source: Materials (Basel). 2022 Feb 24;15(5):1685. doi: 10.3390/ma15051685 (PMC8910993; doi:10.3390/ma15051685)
Supplement: Supplementary file 1 [file materials-15-01685-s001.zip › materials-1568695-supplementary.pdf]

Supporting Information File

Table S1. ICDD Card numbers and crystal structures for the obtained phases.

ICCD card number	Compound name	Mineral name	Crystal structure
**01-083-1413**	Silicon Oxide (SiO_2)	Coesite	Monoclinic
**01-077-8309**	Silicon Oxide (SiO_2)	Cristobalite	Tetragonal
**01-077-8632**	Silicon Oxide (SiO_2)	Cristobalite beta	Cubic
**00-051-1379**	Silicon Oxide (SiO_2)	-	Tetragonal
**05-001-0489**	Silicon Oxide (SiO_2)	Tridymite	Monoclinic
**01-077-8869**	Boron nitride(h-BN)	-	Hexagonal

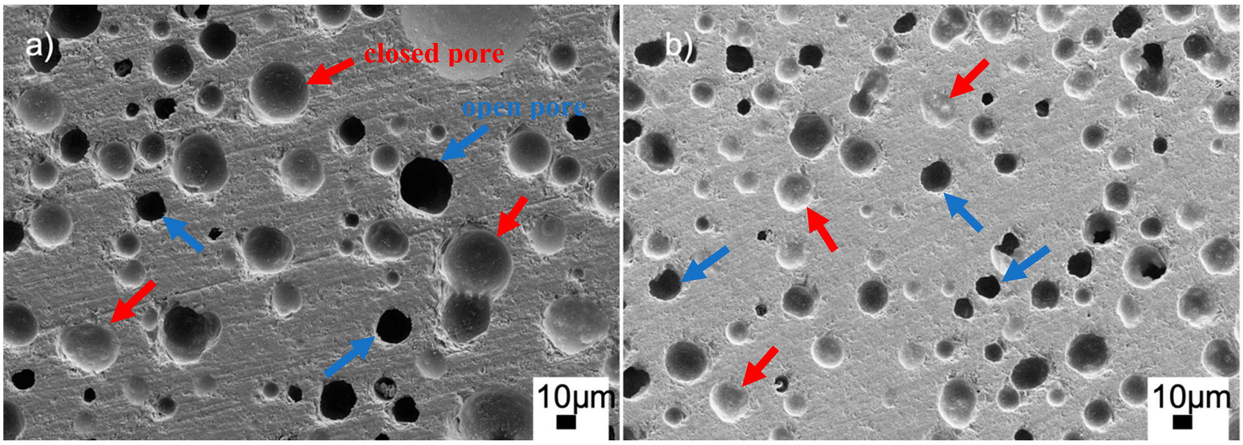

Figure S1. SEM images of sintered glass ceramics at 1.00 KX (**a**) G1PVA@770, (**b**) G3PVA770.

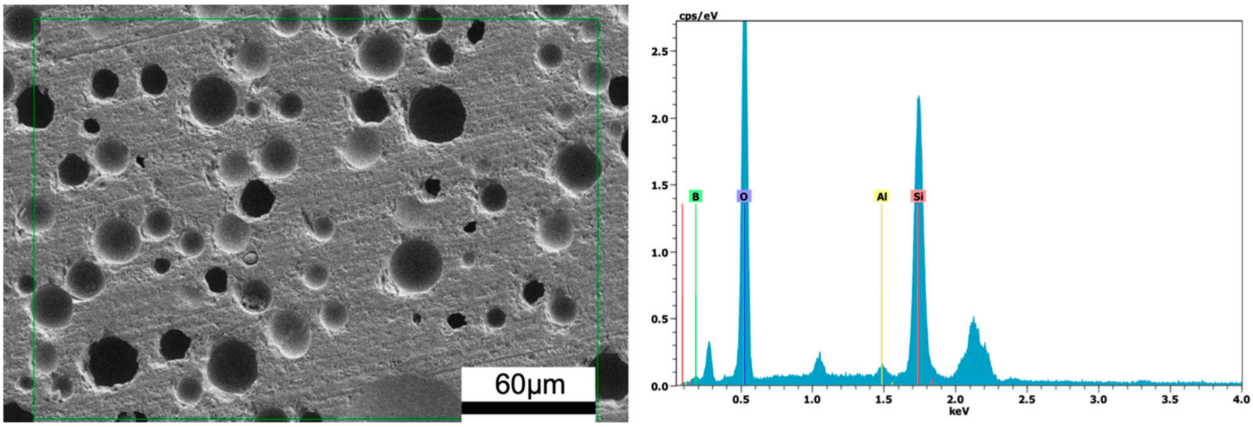

Figure S2. EDS analysis of the G1PVA@770 sample.

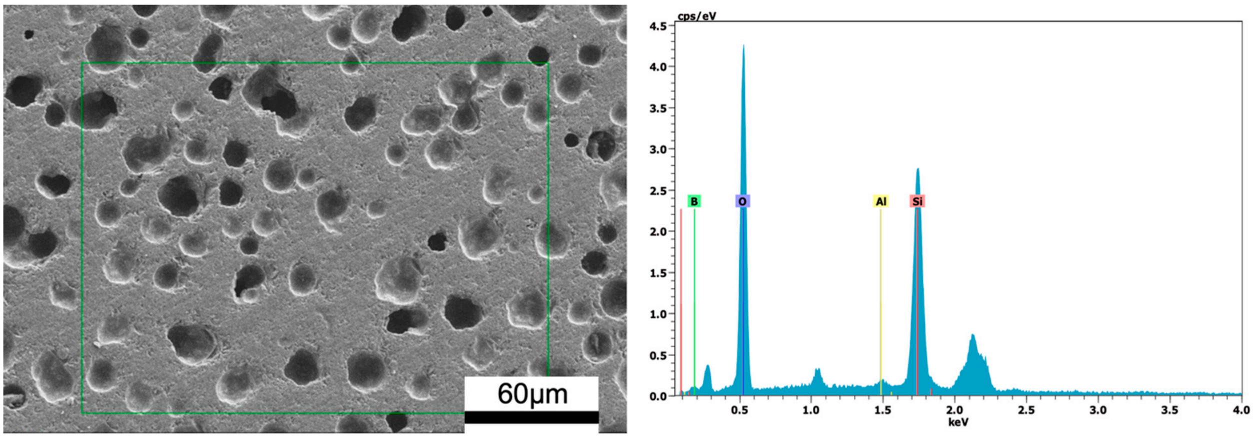

Figure S3. EDS analysis of the G3PVA@770 sample.

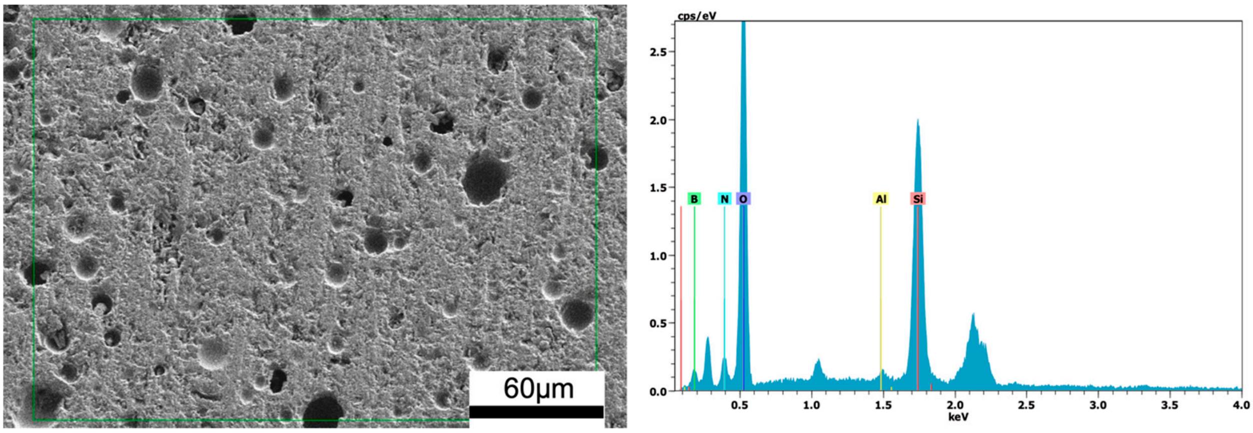

Figure S4. EDS analysis of the G9BN@770 sample.

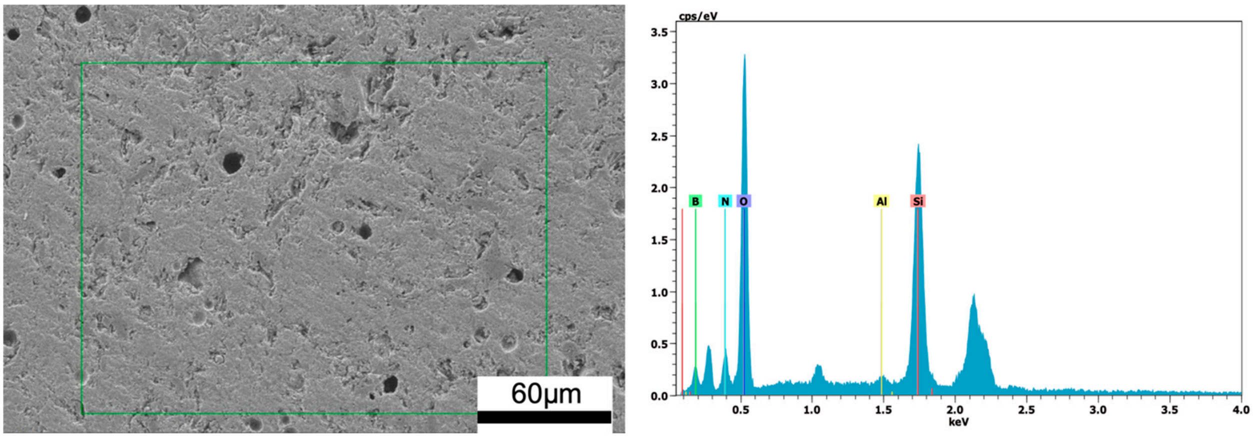

Figure S5. EDS analysis of the G12BN@770 sample.

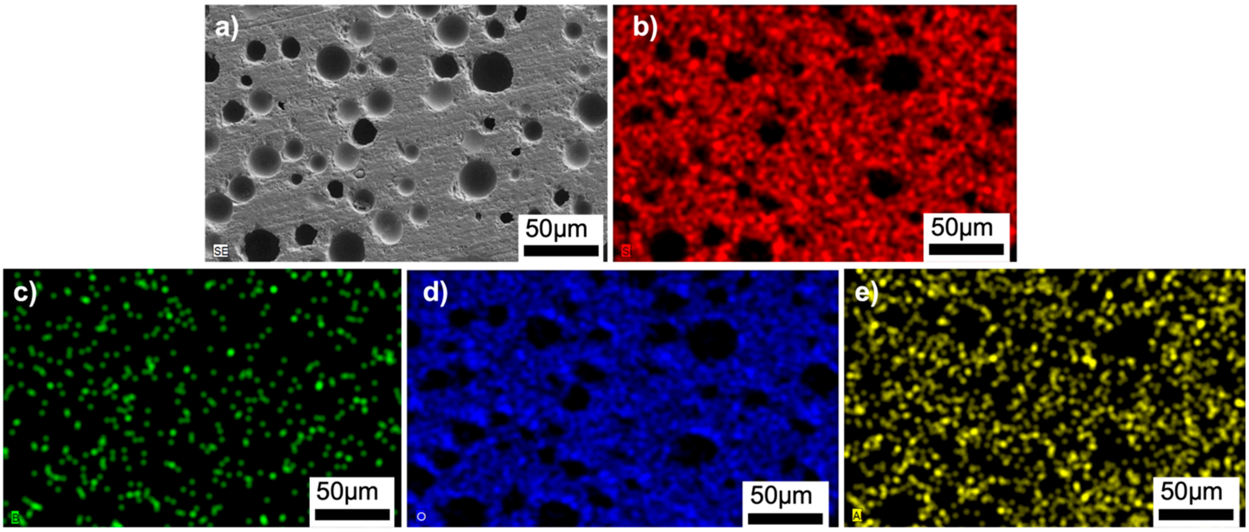

Figure S6. SEM/EDX analyses of the G1PVA@770 sample (**a**) SEM image, (**b**) EDX mapping of Si, (**c**) EDX mapping of B, (**d**) EDX mapping of O, (**e**) EDX mapping of Al.

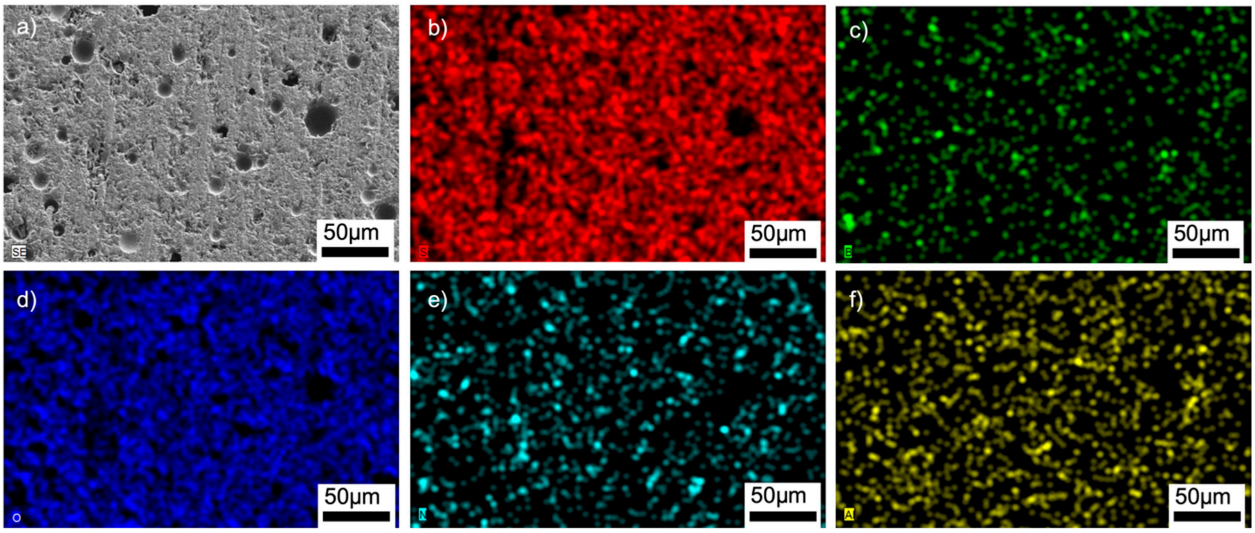

Figure S7. SEM/EDX analyses of the G9BN@770 sample (**a**) SEM image, (**b**) EDX mapping of Si, (**c**) EDX mapping of B, (**d**) EDX mapping of O, (**e**) EDX mapping of Al.
